# Cerebrospinal Fluid Biomarkers in Differential Diagnosis of Multiple Sclerosis and Systemic Inflammatory Diseases with Central Nervous System Involvement

**DOI:** 10.3390/biomedicines11020425

**Published:** 2023-02-01

**Authors:** Mariola Świderek-Matysiak, Magdalena Oset, Małgorzata Domowicz, Grażyna Galazka, Magdalena Namiecińska, Mariusz Stasiołek

**Affiliations:** Department of Neurology, Medical University of Lodz, Kościuszki Street 4, 90-419 Lodz, Poland

**Keywords:** multiple sclerosis, differential diagnosis, cerebrospinal fluid, oligoclonal bands, neurofilaments light chain, interleukin 6, osteopontin

## Abstract

Background: Diagnosis of multiple sclerosis (MS) is established on criteria according to clinical and radiological manifestation. Cerebrospinal fluid (CSF) analysis is an important part of differential diagnosis of MS and other inflammatory processes in the central nervous system (CNS). Methods: In total, 242 CSF samples were collected from patients undergoing differential MS diagnosis because of the presence of T2-hyperintensive lesions on brain MRI. The non-MS patients were subdivided into systemic inflammatory diseases with CNS involvement (SID) or cerebrovascular diseases (CVD) or other non-inflammatory diseases (NID). All samples were analyzed for the presence of oligoclonal bands and ELISA was performed for detection of: INF gamma, IL-6, neurofilaments light chain (NF-L), GFAP, CHI3L1, CXCL13, and osteopontin. Results: The level of IL-6 (*p* = 0.024), osteopontin (*p* = 0.0002), and NF-L (*p* = 0.002) was significantly different among groups. IL-6 (*p* = 0.0350) and NF-L (*p* = 0.0015) level was significantly higher in SID compared to NID patients. A significantly higher level of osteopontin (*p* = 0.00026) and NF-L (*p* = 0.002) in MS compared to NID population was noted. ROC analysis found weak diagnostic power for osteopontin and NFL-L. Conclusions: The classical and non-standard markers of inflammatory process and neurodegeneration do not allow for sufficient differentiation between MS and non-MS inflammatory CNS disorders. Weak diagnostic power observed for the osteopontin and NF-L needs to be further investigated.

## 1. Introduction

Multiple sclerosis (MS) is an autoimmune demyelinating disorder of the central nervous system (CNS) that affects more than two-million people around the world and is mostly diagnosed in patients between 20 and 40 years old. In recent years, numerous studies have shown that early initiation of disease modifying therapy in MS patients is associated with favorable prognosis, and, therefore, emphasis is placed on prompt diagnosis. Diagnosis of MS is established on the 2017 update to the McDonald criteria according to clinical and radiological manifestation [1]. The most important diagnostic tool is magnetic resonance imaging (MRI) with T2-weighted MRI images identifying demyelinating MS lesions [2,3]. However, routine MRI do not specifically detect MS lesions and misdiagnoses were described in many studies. The primary reason for MS misdiagnosis is inappropriate application of McDonald criteria in patients lacking objective clinical findings consistent with MS, and the mistake in interpretation of MRI abnormalities in the non-specific neurologic symptoms [4]. During diagnostic process other diagnoses, such as neuromyelitis optica (NMO) spectrum disorders, conversion/psychogenic disorders, migraine, vascular disease, along with systemic inflammatory diseases with CNS involvement (SID), infectious, and metabolic disorders, should be considered as MS mimics. Systemic inflammatory diseases with CNS involvement, such as lupus, Sjögren’s syndrome, and sarcoidosis due to uncharacteristic clinical symptoms and radiological picture, cause difficulties in differential diagnosis of MS [5,6,7,8,9].

In addition to the MRI examination, cerebrospinal fluid (CSF) analysis is also an important part of the differential MS diagnosis, especially according to current McDonald criteria and the role of the CSF-restricted oligoclonal bands (OCBs) in early diagnosis [2]. CSF analysis with IgG OCBs detection still represents a common step of the diagnostic process, helpful in exclusion of alternative diagnoses. It should be noted that CSF findings typical of MS are not specific to MS. OCBs in CSF have nearly 86% specificity and more than 95% sensitivity for the diagnosis of MS [10]. CSF OCBs detection may significantly support the diagnosis of MS, but only if other causes of CNS inflammation have been ruled out [11]. CSF-restricted OCBs may be elevated in other inflammatory and infectious diseases when clinical and radiological manifestation overlap with MS. The IgG index, known as the Link index, is used to assess a quantitative evaluation of IgG intrathecal synthesis; however, it has been defined that the Link index is less sensitive than detection of CSF OCBs [10,11,12]. 

Specific biomarkers or sets of biomarkers in MS diagnostic process are still lacking. In MS pathology both inflammatory and degenerative processes occur during the early phase of the disease. Among the recently proposed diagnostic tests is kappa free light chains index [13,14] and measles-rubella varicella-zoster (MRZ) reaction, both of which have been shown an intermediate specificity as diagnostic biomarkers requiring further confirmatory studies [15,16]. Neurofilaments are cytoskeletal proteins released from damaged axons into the CSF and the blood, and are considered biomarkers of neurodegeneration process. Neurofilaments light chain (NF-L) have been elevated in CSF and blood of patients with many neurodegenerative disorders, including MS. NF-L and glial fibrillary acidic protein (GFAP) have a confirmed prognostic value in MS but low diagnostic value [17,18,19]. Chitinase-3-like-1 (CHI3L1; YKL-40a) [20], osteopontin (OPN) [21,22,23] and C-X-C motif ligand 13 (CXCL13) [24,25] are the markers of the inflammatory process and have been mainly suggested as disease activity and treatment response biomarkers. While CHI3L1, OPN, and CXCL13 levels in CSF might be helpful in diagnostic process of patients suspected of MS, those markers require further validation.

Diagnostic biomarkers should be able to improve the accuracy of the diagnosis when applied together with clinical and MRI criteria. The aim of this study was to evaluate the diagnostic value of non-standard CSF biomarkers: interferon gamma (IFN-γ), interleukin 6 (IL6), NF-L, GFAP, CHI3L1, CXCL13, and OPN in patients suspected of MS according to the presence of T2-hyperintensive lesions on brain MRI.

## 2. Material and Methods

### 2.1. Patient Selection

This prospective study included 333 patients undergoing differential MS diagnosis because of the presence of T2-hyperintensive lesions on brain MRI suggestive of demyelinating lesions. We prospectively collected CSF samples during diagnostic procedures from 242 patients hospitalized in the Department of Neurology Medical University of Lodz, Barlicki Hospital, between July 2018 and June 2021. The study was conducted according to the guidelines of the Declaration of Helsinki (1964) and its later amendments and received approval of the Local Ethics Committee of the Medical University of Lodz (approval number/360/17/KE, 21 November 2017, RNN/231/18KE, 12 June 2018). Informed consent was obtained from all the subjects involved in the study.

Patients had to be 18–55 years old and had T2-hyperintensive lesions on brain MRI. MS diagnosis was made according to the 2017 McDonald criteria [2]. The non-MS patients were subdivided into several diagnostic groups classified as systemic inflammatory diseases with CNS involvement (SID) or cerebrovascular diseases (CVD) or other non-inflammatory diseases (NID). The participants were without steroid treatment within at least 3 months before enrolment into the study and had no history of any other immunomodulatory or immunosuppressive treatment. 

### 2.2. IFN-γ, CXCL13, GFAP, CHI3L1, OPN, IL6 and NF-Light Assessment in CSF

The same sample collection procedure was applied to all participants. CSF samples were stored at −80 °C before use. All samples were analyzed for the presence of OCBs through CSF and serum immunoelectrophoresis in the same certified laboratory. Concentrations of human CXCL13, CHI3L1, OPN, IL6, and IFN gamma (Biorbyt Ltd. (Cambridge, UK)), GFAP (Wuhan EIAab Science (Wuhan, China)), and NF-L (Uman Diagnostics (Umea, Sweden)) were measured in CSF using appropriate ELISA kits. All reagents were used according to the manufacturer’s recommendations. 

Briefly, for CXCL13, CHI3L1, OPN, IL6, IFN-γ, GFAP, and NF-L measurements CSF samples were applied on plates coated with specific antibody against tested antigen, respectively, and molecular agent present in a sample was bound to the wells. After washing and adding biotinylated antihuman antibody against tested factor and HRP-conjugated streptavidin and TMB substrate solution, the intensity of the developed color was measured at 450 nm on EPOCH (BioTek (Tokyo, Japan)) microplate reader. The intra-assay validation was performed and four quality control (QC) samples were included on each plate. All assays were run in duplicates and the average percentage coefficient of variation (CV) was 15% or less. The catalog/lot number of used ELISA kits (Table S1) and the representative standard curves (Figure S1) are available in the Supplementary Materials.

Detection limits for measured antigens were 1 pg/mL for CXCL13, 10 pg/mL for CHI3L1, 10 pg/mL for OPN, 0.3 pg/mL for IL6, 2 pg/mL for IFN gamma, 0.12 ng/mL for GFAP, and 33 pg/mL for NF-L. Due to the concentration of GFAP below the detection threshold, it was not included in the analysis.

### 2.3. Statistic

Analyses were performed with IBM SPSS Statistics 28 software. Due to the violation of assumptions of normal data distribution and homogeneity of variance for all biomarkers, as revealed by Shapiro–Wilk test and Levene’s test, respectively, non-parametrical tests were implemented. Kruskal–Wallis test was used to assess the differences in levels of biomarkers among study groups. The Bonferroni correction for multiple testing was used when appropriate.

Spearman rank correlation was performed to analyze the associations between the biomarkers and to test the relation between the levels of biomarkers and age. In linear regression models all of the biomarkers proved not to be dependent of age, so we did not perform normalization for this variable. A receiver operating characteristic (ROC) curve, calculation of area under the curve (AUC) and its 95% confidence interval (CI) was used to plot the true positive rate vs. the false positive rate of the biomarkers. The best cut-off score was selected based on the value that maximized sensitivity and specificity at the same time. Finally, sensitivity, specificity, and positive and negative likelihood ratios (LRs) were calculated for the best cut-off value of biomarkers level.

## 3. Results

### 3.1. Study Population and Standard OCBs Analysis

Demographic and medical data of all the participants were collected from a local medical database. After final diagnosis had been made patients were divided into four groups for further analysis:

(1) 134 patients with MS (PwMS) according to McDonald criteria 2017;
(2) 28 patients with systemic inflammatory diseases with CNS involvement (SID);
(3) 10 patients with cerebrovascular diseases (CVD) (e.g., small vessel disease);
(4) 70 patients with other non-inflammatory diseases (NID) (e.g., migraine, depression, patients with MRI non-specific white matter lesions).

The majority of patients with SID were diagnosed with systemic lupus erythematosus (SLE) (11 patients). Other diagnoses in this group were: undifferentiated connective tissue disease (six patients patients), rheumatoid arthritis (four patients), CNS vasculitis (three patients), Sjögren’s syndrome (one patient), neurosarcoidosis (three patients). Demographic data and standard OCBs assessment results are reported in Table 1. 

OCBs specific for CSF were detected in most PwMS (93.3%), whereas non-MS patients were characterized by significantly lower frequency of CSF specific OCBs—28.6%, 5.7% and 0% in SID, NID, and CVD groups, respectively. In the analysis of the whole study population, sensitivity and specificity of OCBs assessment in MS diagnosis was 91.2% and 89.2%, respectively. Sensitivity of OCBs in the diagnosis of CNS inflammatory diseases (pooled PwMS and SID population) was also high but specificity lower to 70.4% (Table 2).

### 3.2. Assessment of Non-Standard CSF Biomarkers 

Because of the significant age differences between particular diagnostic groups (in post hoc analysis MS v. CVD *p* = 0.037; MS v. NID *p* = 0.099) statistical analysis was performed in order to adjust for age. However, in linear regression model levels of biomarkers proved to be independent of age. Moreover, we did not find any significant correlation between age and levels of any biomarker in CSF samples collected in the study (CXCL13 *p* = 0.325, CHI3L1 *p* = 0.569, OPN, *p* = 0.254, IL6 *p* = 0.599, INF-γ *p* = 0.420, NF-L *p* = 0.315). 

Spearman rank correlation was performed to analyze the associations between the concentrations of the investigated non-standard CSF biomarkers. In the whole study group we found only weak positive association between IL6 and CHI3L1 level (R = 0.29, *p* = 0.000) and OPN and NF-L (R = 0.25, *p* = 0.000), and weak negative association between INF-γ and CHI3L1 (R = −0.21, *p* = 0.001) and OPN and CXCL13 (R = −0.18, *p* = 0.005) (Table 3).

**Figure 1:**
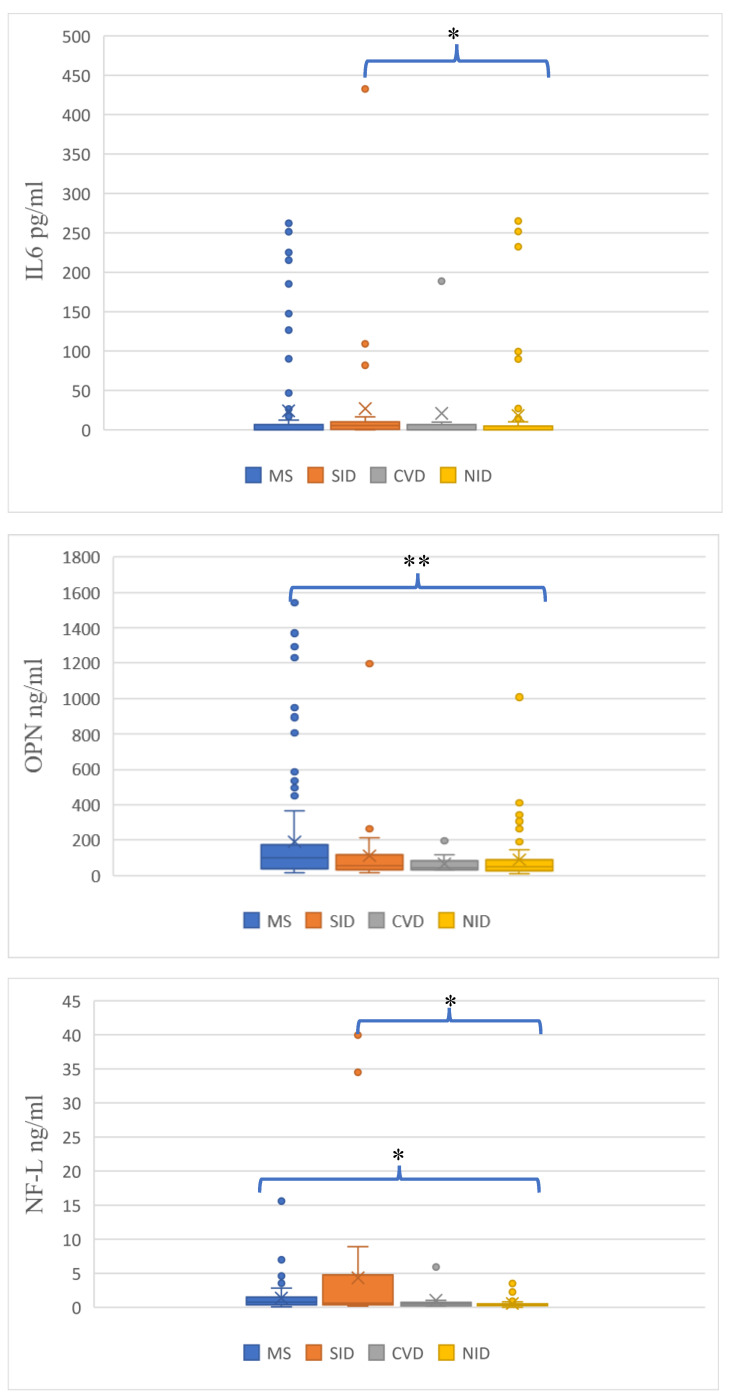
CSF concentration of IL6, OPN and NF-L. IL-6 (*p* = 0.035) and NF-L (*p* = 0.002) level was significantly higher in SID compared to NID patients. Significantly higher level of OPN (*p* = 0.000) and NF-L (*p* = 0.002) in MS compared to NID population was noted. * means significant differences *p* < 0.05; ** means significant differences *p* < 0.001.

We noted higher mean concentration of INF-γ and CXCL13 in SID group but there was no significant difference among study groups (Table 4). The level of IL6 was significantly different among groups (*p* = 0.024), and post hoc analysis revealed that IL6 concentration in CSF was significantly higher in SID as compared to NID patients (*p* = 0.035). The concentration of OPN showed significant differences among groups (*p* = 0.000), post hoc analysis revealed significantly higher level of OPN in PwMS in comparison to NID population (*p* = 0.000). Furthermore, levels of NF-L differed significantly among study groups (*p* = 0.002). The higher mean concentration of NF-L was observed in SID patients. Post hoc analysis found significantly different levels of NF-L between SID and NID patient populations (*p* = 0.002) and also between PwMS and NID group (*p* = 0.002) (Figure 1). 

**Figure 2:**
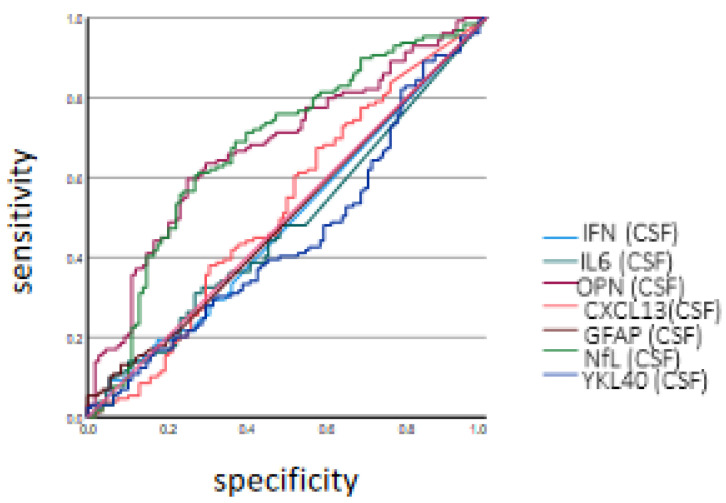
ROC analysis found statistically significant AUC for OPN and NFL-L. AUC was 0.68 for both OPN and NF-L (CI 95% 0.6–0.7, *p* < 0.005 for both biomarkers).

In the final step, we performed ROC curve analysis to evaluate the diagnostic utility of non-standard CSF biomarkers in MS diagnosis. ROC analysis found statistically significant AUC for OPN and NF-L, but with weak diagnostic power. AUC was 0.68 for both OPN and NF-L (CI 95% 0.6–0.7, *p* < 0.05 for both biomarkers) (Figure 2). In a subsequent analysis, a CSF NF-L value of 0.428 ng/mL was the cut-off diagnostic point with a sensitivity of 70.4% and specificity of 58.0%, with a Positive LR of 1.68 and a Negative LR of 0.51. We also determined an optimal cut-off value of 64.746 ng/mL for OPN, with a sensitivity of 63.7% and specificity of 60.7%, with a Positive LR of 1.62 and a Negative LR of 0.59.

## 4. Discussion

Using the 2017 McDonald criteria, MS is diagnosed in a patient presenting with appropriate symptoms and evidence of one or more demyelinating lesion in CNS. The differential diagnosis of MS is based on the typical clinical, radiological and laboratory findings together with the exclusion of other disorders which could better explain the clinical presentation in particular patients. However, none of the parameters used in the diagnostic process is specific for MS [2], which represents a very important factor, especially in cases with rare, atypical, or very mild clinical presentations. The diagnostic challenges have been in the last years additionally confronted with the constantly increasing need of rapid diagnostic and therapeutic decisions in MS [26], which may, in turn, lead to misdiagnoses and all the negative consequences for the patients. Since the misinterpretation of MRI T2-hyperintensive lesions belongs to the main confounders, the diagnosis of MS should not be based solely on the MRI findings [6,27]. The history of other inflammatory and/or autoimmune diseases, including SID, seems to be another very important factor contributing to the misdiagnosis of MS [6,9,28]. Diagnosis of even the most common SID, such as lupus, undifferentiated connective tissue disease, or Sjögren’s syndrome, is difficult due to the rich and often uncharacteristic symptomatology. 

In our study, we attempted to analyze the applicability of CSF molecular biomarkers in the differential diagnosis of MS. In a relatively large group (n = 333) of prospectively recruited patients undergoing neurological diagnosis because of the brain MRI findings suggestive of demyelinating lesions, we confirmed that the presence of OCBs in the initial CSF examination was characterized by high sensitivity and specificity for MS diagnosis [2,10,29]. The sensitivity of OCB remained high in the analysis of the combined groups of patients with inflammatory CNS conditions (MS and SID); however, the specificity was much lower. While much lower than in MS, a still relatively high frequency of OCB in SID patients in our study stays in line with the recently published findings regarding systemic inflammatory diseases with CNS involvement [30,31,32] and non-MS inflammatory conditions [33]. In the further analysis non-standard molecular CSF biomarkers of various aspects of inflammatory reaction (CXCL13, IL6, IFN-γ, CHI3L1, OPN), glial (GFAP), and axonal damage or neurodegeneration (NF-L) [34,35,36,37] were investigated. Although the average age differed significantly between study groups, statistical analysis showed that the CSF levels of biomarkers were independent of age in our study. This is of great importance, since processes associated with normal aging may influence levels of both inflammatory [38] as well as structural biomarkers [18]. The lack of such age dependent changes in biomarker levels in our group may result from the relatively narrow age range and/or insufficient numbers of patients to detect slight changes associated with aging. 

Many previous reports demonstrated increased NF-L concentrations in CSF of MS patients at different stages of the disease [39,40,41]. In accordance, in our study we detected significantly higher CSF levels of NF-L in MS as compared to the NID group. However, such difference was also observed between SID and NID patients, whereas the NF-L levels were similar in both inflammatory groups—MS and SID—underscoring the well-known, unspecific nature of NF-L increase [36]. Importantly, elevated serum and CSF levels of NF-L were reported in various SID (including lupus, sarcoidosis, Sjögren’s syndrome), both in patients with [42,43,44] and without neuro-psychiatric presentation [45]. Our analysis also revealed increased CSF concentration of OPN in PwMS in comparison to NID population. This observation stays in agreement with numerous other studies reporting elevated OPN levels in CSF and/or peripheral blood of MS patients as compared to healthy controls, and non-inflammatory neurological diseases [23]. Noteworthily, in our study the CSF concentration of OPN was similar in patients with MS and SID, which supports earlier findings in other groups of various SID patients [21,22]. However, there are also reports with contradictory findings, which may result from a variety of reasons, including different diseases classified as inflammatory CNS conditions in particular studies, differences of age and comorbidity structure [23,46]. Another observation of this study is the significantly higher CSF concentration of IL6 in SID as compared to NID patients. In concordance with our results, CSF IL6 is considered a major biomarker of neuro-psychiatric lupus [47]. Elevated CSF levels of this cytokine has also been demonstrated in other inflammatory CNS diseases, such as neurosarcoidosis [44,48], and mixed groups, including NMOSD, CNS vasculitis, neurosarcoidosis, and neuroinfection [49]. However, the limited data available regarding the comparison of IL6 in SID and MS patients do not allow for unequivocal conclusions and indicate rather significant dependence on particular inflammatory condition [48,49,50].

In our study we did not find significant differences between patient groups in CSF concentrations of INF-γ, CXCL13, and CHI3L1. Recent meta-analysis of research data based on over 200 studies with cumulative number of ca. 13,500 MS patients indicated CSF levels of CXCL13 and INF-γ as, respectively, strong and moderate differentiating biomarkers of MS [24], which also finds support in later studies assessing multiple molecular factors in MS [51]. Such discrepancy may possibly be associated with dissimilar demographic characteristics of both MS and control non-MS groups, as well as different clinical status of MS patients, including the stage of the disease and treatment history. Additionally, both cytokines are considered as not specific for MS, and their CSF levels may be elevated also in other inflammatory CNS diseases, including neuroinfection [52,53]. Similarly, increased concentration of CHI3L1 in CSF has been reported in MS patients [54], however the protein seems to be not specific for MS and its CSF concentration may be higher in other inflammatory CNS conditions [55]. The most recently published study evaluating multiple CSF molecules in a group of patients with different clinical forms of MS and other inflammatory and non-inflammatory neurological disorders suggested CSF CXCL13 and CHI3L1 levels as very good prognostic biomarkers in relapsing MS patients, including CIS conversion to definitive MS and clinical and radiological disease activity [56]. Thus, we may again speculate that the lack of differences in CSF concentrations of these molecules between MS and non-MS patients in our study reflects the very early stage of MS in our patients and could be additionally associated with the wide association of this markers with MS and other inflammatory CNS diseases.

Due to the low specificity of the 2017 McDonald criteria in the diagnosis of MS, radiological, clinical, and molecular markers that would help clinicians in making an early diagnosis and minimizing the risk of misdiagnosis are being searched for. In the near future, an artificial intelligence approach can be a diagnostic tool that in a multiparametric analysis will take into account MS-specific brain and spinal cord MRI features, combined with clinical symptoms and blood/CSF markers, and will help clinicians in the most probable diagnosis [57].

The main limitation of our study is the relative low number of patients in the non-MS groups, which is, however, directly associated with the routine indications for lumbar puncture and CSF examination. It must be underlined that numerous studies published so far were affected by similar imbalance in the quantity of MS and control groups. Another limitation of this study is the lack of longitudinal observation, although the aim of the study was to evaluate diagnostic utility of selected biomarkers, again with the use of CSF obtained at initial workup of the patient. 

## 5. Conclusions

Taken together, the results of our study performed in the prospectively recruited group of patients with various CNS disorders suggest that the selected biomarkers representing both classical and non-standard markers of inflammatory process and neurodegeneration do not allow for sufficient differentiation between MS and non-MS inflammatory CNS disorders. However, weak diagnostic power observed for the OPN and NF-L in CSF needs to be investigated further in larger and more homogenous groups of patients with systemic inflammatory diseases with CNS involvement.

## Figures and Tables

**Table 1:** Patient demographics.

Patients n = 242	MS n = 134	SID n = 28	CVD n = 10	NID n = 70
Female sex, n (%)	97 (72.4)	17 (60.7)	7 (70.0)	58 (82.8)
Age, years	35.8 (10.4) *	39.3 (10.3)	44.3 (7.9) *	42.4 (12.9) *
OCBs, n (%)	125 (93.3) *	8 (28.6) *	0 (0) *	4 (5.7) *

All values are reported as mean (standard deviation) unless indicated otherwise. MS, multiple sclerosis; SID, systemic inflammatory diseases with CNS involvement; CVD, cerebrovascular diseases; NID, other non-inflammatory diseases; OCBs, oligoclonal bands. * means significant differences *p* < 0.05.

**Table 2:** Diagnostic sensitivity and specificity of OCBs.

	Patients with T2 Lesions in MRI Suspected of MS	PwMS and SID Population
OCBs sensitivity	91.2%	93.9%
OCBs specificity	89.2%	70.4%

PwMS, patients with multiple sclerosis; SID, systemic inflammatory diseases with CNS involvement; OCBs, oligoclonal bands.

**Table 3:** Relationships between CSF biomarkers.

Biomarkers	Spearman R	*p*
INF-γ and CXCL13	0.132640	0.037
INF-γ and CHI3L1	−0.205150	0.001 *
IL6 and CHI3L1	0.297844	0.000 *
OPN and CXCL13	−0.179078	0.005 *
OPN and NF-L	0.256492	0.000 *
OPN and IL6	−0.126423	0.047

IFN-γ, interferon gamma; NF-L, neurofilaments light chain; CHI3L1, chitinase-3-like-1; OPN, osteopontin; * means significant differences *p* < 0.05.

**Table 4:** CSF biomarkers concentration.

Patients n = 242	MS n = 134	SID n = 28	CVD n = 10	NID n = 70
INF-γ (pg/mL)	9.9 (43.6) 0.0 [0.0–5.2]	29.3 (87.4) 0.9 [0.0–9.9]	12.9 (23.7) 1.2 [0.0–16.1]	4.7 (10.8) 0.0 [0.0–4.6]
IL6 (pg/mL)	23.9 (64.7) 0.0 [0.0–6.4]	26.9 (83.1) 5.5 [1.2–10.0]	20.9 (59.1) 0.0 [0.0–5.7]	18.2 (57.4) 0.0 [0.0–4.6]
OPN (ng/mL)	189.1 (274.9) 95.3 [37.9–170.7]	112.2 (221.2) 53.4 [30.9–108.4	66.1 (52.9) 43.2 [32.8–67.6]	89.5 (134.8) 51.5 [28.8–86.6]
CXCL13 (pg/mL)	260.7 (250.3) 207.8 [71.2–397.6]	411.2 (802.3) 129.4 [0.0–452.4]	295.6 (359.9) 157.7 [62.5–301.9]	260.0 (251.9) 220.9 [33.4–418.5]
NF-L (ng/mL)	1.4 (2.1) 0.8 [0.4–1.5]	4.3 (9.6) 0.6 [0.4–3.9]	1.0 (1.7) 0.5 [0.3–0.6]	0.6 (0.6) 0.3 [0.3–0.5]
CHI3L1 (ng/mL)	32.9 (63.3) 10.9 [5.7–29.8]	33.4 (48.9) 27.5 [6.7–38.1]	39.2 (69.9) 21.5 [7.5–28.8]	38.9 (59.5) 27.1 [6.8–33.8]

All values reported as mean (standard deviation) in the first row and median [Q1–Q3] in the second row. IFN-γ, interferon gamma; NF-L, neurofilaments light chain; CHI3L1, chitinase-3-like-1; OPN, osteopontin.

## Data Availability

The datasets analyzed or generated during the current study are available from the corresponding author upon reasonable request.

## Supplementary Materials

The following supporting information can be downloaded at: https://www.mdpi.com/article/10.3390/biomedicines11020425/s1, Figure S1: The representative standard curves of used ELISA kits.; Table S1: The catalog/lot number of used ELISA kits.

## References

[1] Oh J., Vidal-Jordana A., Montalban X. (2018). Multiple sclerosis: Clinical aspects. Curr. Opin. Neurol..

[2] Thompson A.J., Banwell B.L., Barkhof F., Carroll W.M., Coetzee T., Comi G., Correale J., Fazekas F., Filippi M., Freedman M.S. (2018). Diagnosis of multiple sclerosis: 2017 revisions of the McDonald criteria. Lancet Neurol..

[3] Wattjes M.P., Ciccarelli O., Reich D.S., Banwell B., de Stefano N., Enzinger C., Fazekas F., Filippi M., Frederiksen J., Gasperini C. (2021). Magnetic Resonance Imaging in Multiple Sclerosis Study Group; Consortium of Multiple Sclerosis Centres; North American Imaging in Multiple Sclerosis Cooperative MRI guidelines working group. 2021 MAGNIMS-CMSC-NAIMS consensus recommendations on the use of MRI in patients with multiple sclerosis. Lancet Neurol..

[4] Solomon A.J., Naismith R.T., Cross A.H. (2019). Misdiagnosis of multiple sclerosis: Impact of the 2017 McDonald criteria on clinical practice. Neurology.

[5] Solomon A.J., Bourdette D.N., Cross A.H., Applebee A., Skidd P.M., Howard D.B., Spain R.I., Cameron M.H., Kim E., Mass M.K. (2016). The contemporary spectrum of multiple sclerosis misdiagnosis: A multicenter study. Neurology.

[6] Midaglia L., Sastre-Garriga J., Pappolla A., Quibus L., Carvajal R., Vidal-Jordana A., Arrambide G., Río J., Comabella M., Nos C. (2021). The frequency and characteristics of MS misdiagnosis in patients referred to the multiple sclerosis centre of Catalonia. Mult. Scler..

[7] Wildner P., Stasiołek M., Matysiak M. (2020). Differential diagnosis of multiple sclerosis and other inflammatory CNS diseases. Mult. Scler. Relat. Disord..

[8] Gaitán M.I., Sanchez M., Farez M.F., Fiol M.P., Ysrraelit M.C., Solomon A.J., Correale J. (2022). The frequency and characteristics of multiple sclerosis misdiagnosis in Latin America: A referral center study in Buenos Aires, Argentina. Mult. Scler..

[9] Kaisey M., Solomon A.J., Luu M., Giesser B.S., Sicotte N.L. (2019). Incidence of multiple sclerosis misdiagnosis in referrals to two academic centers. Mult. Scler. Relat. Disord..

[10] Arrambide G., Tintore M., Espejo C., Auger C., Castillo M., Vidal-Jordana A., Galán I., Nos C., Mitjana R., Mulero P. (2018). The value of oligoclonal bands in the multiple sclerosis diagnostic criteria. Brain.

[11] Freedman M.S., Thompson E.J., Deisenhammer F., Giovannoni G., Grimsley G., Keir G., Ohman S., Racke M.K., Sharief M., Sindic C.J. (2005). Recommended standard of cerebrospinal fluid analysis in the diagnosis of multiple sclerosis: A consensus statement. Arch. Neurol..

[12] Mayringer I., Timeltaler B., Deisenhammer F. (2005). Correlation between the IgG index, oligoclonal bands in CSF, and the diagnosis of demyelinating diseases. Eur. J. Neurol..

[13] Konen F.F., Schwenkenbecher P., Jendretzky K.F., Gingele S., Sühs K.-F., Tumani H., Süße M., Skripuletz T. (2021). The Increasing Role of Kappa Free Light Chains in the Diagnosis of Multiple Sclerosis. Cells.

[14] Leurs C.E., Twaalfhoven H., Lissenberg-Witte B.I., van Pesch V., Dujmovic I., Drulovic J., Castellazzi M., Bellini T., Pugliatti M., Kuhle J. (2020). Kappa free light chains is a valid tool in the diagnostics of MS: A large multicenter study. Mult. Scler..

[15] Felgenhauer K., Reiber H. (1992). The diagnostic significance of antibody specificity indices in multiple sclerosis and herpes virus induced diseases of the nervous system. Clin. Investig..

[16] Jarius S., Eichhorn P., Franciotta D., Petereit H.F., Akman-Demir G., Wick M., Wildemann B. (2017). The MRZ reaction as a highly specific marker of multiple sclerosis: Re-evaluation and structured review of the literature. J. Neurol..

[17] Norgren N., Sundström P., Svenningsson A., Rosengren L., Stigbrand T., Gunnarsson M. (2004). Neurofilament and glial fibrillary acidic protein in multiple sclerosis. Neurology.

[18] Bridel C., van Wieringen W.N., Zetterberg H., Tijms B.M., Teunissen C.E., Alvarez-Cermeño C.J., Andreasson U., Axelsson M., Bäckström D.C., The NFL Group (2019). Diagnostic value of cerebrospinal fluid neurofilament light protein in neurology: A systematic review and meta-analysis. JAMA Neurol..

[19] Martínez M.A., Olsson B., Bau L., Matas E., Cobo Calvo Á., Andreasson U., Blennow U., Romero-Pinel L., Martínez-Yélamos S., Zetterberg H. (2015). Glial and neuronal markers in cerebrospinal fluid predict progression in multiple sclerosis. Mult. Scler..

[20] Correale J., Fiol M. (2011). Chitinase effects on immune cell response in neuromyelitis optica and multiple sclerosis. Mult. Scler..

[21] Braitch M., Nunan R., Niepel G., Edwards L.J., Constantinescu C.S. (2008). Increased osteopontin levels in the cerebrospinal fluid of patients with multiple sclerosis. Arch. Neurol..

[22] Chowdhury S.A., Lin J., Sadiq S.A. (2008). Specificity and correlation with disease activity of cerebrospinal fluid osteopontin levels in patients with multiple sclerosis. Arch. Neurol..

[23] Agah E., Zardoui A., Saghazadeh A., Ahmadi M., Tafakhori A., Rezaei M. (2018). Osteopontin (OPN) as a CSF and blood biomarker for multiple sclerosis: A systematic review and meta-analysis. PLoS ONE.

[24] Bai Z., Chen D., Wang L., Zhao Y., Liu T., Yu Y., Yan T., Cheng Y. (2019). Cerebrospinal fluid and blood cytokines as biomarkers for multiple sclerosis: A systematic review and meta-analysis of 226 studies with 13,526 multiple sclerosis patients. Front. Neurosci..

[25] Sellebjerg F., Börnsen L., Khademi M., Krakauer M., Olsson T., Frederiksen J.L., Sørensen P.S. (2009). Increased cerebrospinal fluid concentrations of the chemokine CXCL13 in active MS. Neurology.

[26] Brownlee W., Solomon A.J. (2021). Misdiagnosis of multiple sclerosis: Time for action. Mult. Scler..

[27] Brownlee W.J. (2018). Use (and misuse) of the McDonald criteria to diagnose multiple sclerosis. Eur. J. Neurol..

[28] Calabrese M., Gasperini C., Tortorella C., Schiavi G., Frisullo G., Ragonese P., Fantozzi R., Prosperini L., Annovazzi P., Cordioli C. (2019). “Better explanations” in multiple sclerosis diagnostic workup: A 3-year longitudinal study. Neurology.

[29] Dobson R., Ramagopalan S., Davis A., Giovannoni G. (2013). Cerebrospinal fluid oligoclonal bands in multiple sclerosis and clinically isolated syndromes: A meta-analysis of prevalence, prognosis and effect of latitude. J. Neurol. Neurosurg. Psychiatry.

[30] Karathanasis D., Rapti A., Nezos A., Skarlis C., Kilidireas C., Mavragani C.P., Evangelopoulos M.E. (2022). Differentiating central nervous system demyelinating disorders: The role of clinical, laboratory, imaging characteristics and peripheral blood type I interferon activity. Front. Pharmacol..

[31] Nikolopoulos D., Kitsos D., Papathanasiou M., Chondrogianni M., Theodorou A., Garantziotis P., Pieta A., Doskas T., Bertsias G., Voumvourakis K. (2021). Demyelination with autoimmune features: A distinct clinical entity? Results from a longitudinal cohort. Rheumatology.

[32] Nikolopoulos D., Kitsos D., Papathanasiou M., Kapsala N., Garantziotis P., Pieta A., Gioti O., Grivas A., Voumvourakis K., Boumpas D. (2022). Demyelinating Syndromes in Systemic Lupus Erythematosus: Data From the “Attikon” Lupus Cohort. Front. Neurol..

[33] Saadeh R.S., Bryant S.C., McKeon A., Weinshenker B., Murray D.L., Pittock S.J., Willrich M.A.V. (2022). CSF Kappa Free Light Chains: Cutoff Validation for Diagnosing Multiple Sclerosis. Mayo. Clin. Proc..

[34] Harris V.K., Tuddenham J.F., Sadiq S.A. (2017). Biomarkers of multiple sclerosis: Current findings. Degener. Neurol. Neuromuscul. Dis..

[35] Ziemssen T., Akgün K., Brück W. (2019). Molecular biomarkers in multiple sclerosis. J. Neuroinflamm..

[36] Kaisey M., Lashgari G., Fert-Bober J., Ontaneda D., Solomon A.J., Sicotte N.L. (2022). An Update on Diagnostic Laboratory Biomarkers for Multiple Sclerosis. Curr. Neurol. Neurosci. Rep..

[37] Saadeh R.S., Ramos P.A., Algeciras-Schimnich A., Flanagan E.P., Pittock S.J., Willrich M.A. (2022). An Update on Laboratory-Based Diagnostic Biomarkers for Multiple Sclerosis and Beyond. Clin. Chem..

[38] Picón C., Tejeda-Velarde A., Fernández-Velasco J.I., Comabella M., Álvarez-Lafuente R., Quintana E., Sainz de la Maza S., Monreal E., Villarrubia N., Álvarez-Cermeño J.C. (2021). Identification of the Immunological Changes Appearing in the CSF During the Early Immunosenescence Process Occurring in Multiple Sclerosis. Front. Immunol..

[39] Lycke J.N., Karlsson J.E., Andersen O., Rosengren L.E. (1998). Neurofilament protein in cerebrospinal fluid: A potential marker of activity in multiple sclerosis. J. Neurol. Neurosurg. Psychiatry.

[40] Malmeström C., Haghighi S., Rosengren L., Andersen O., Lycke J. (2003). Neurofilament light protein and glial fibrillary acidic protein as biological markers in MS. Neurology.

[41] Olesen M.N., Soelberg K., Debrabant B., Nilsson A.C., Lillevang S.T., Grauslund J., Brandslund I., Madsen J.S., Paul F., Smith T.J. (2019). Cerebrospinal fluid biomarkers for predicting development of multiple sclerosis in acute optic neuritis: A population-based prospective cohort study. J. Neuroinflamm..

[42] Lauvsnes M.B., Zetterberg H., Blennow K., Kvaløy J.T., Tjensvoll A.B., Maroni S., Beyer M.K., Greve O.J., Kvivik I., Alves G. (2022). Neurofilament light in plasma is a potential biomarker of central nervous system involvement in systemic lupus erythematosus. J. Neurol..

[43] Tjensvoll A.B., Lauvsnes M.B., Zetterberg H., Kvaløy J.T., Kvivik I., Maroni S.S., Greve O.J., Beyer M.K., Hirohata S., Putterman C. (2021). Neurofilament light is a biomarker of brain involvement in lupus and primary Sjögren’s syndrome. J. Neurol..

[44] Byg K.E., Nielsen H.H., Sejbaek T., Madsen J.S., Olsen D.A., Nguyen N., Kindt A., Grauslund J., Illes Z., Ellingsen T. (2021). Elevated Neurofilament Light Chain in Cerebrospinal Fluid and Plasma Reflect Inflammatory MRI Activity in Neurosarcoidosis. Brain Sci..

[45] Zervides K.A., Janelidze S., Nystedt J., Gullstrand B., Nilsson P., Sundgren P.C., Bengtsson A.A., Hansson O., Jönsen A. (2022). Plasma and cerebrospinal fluid neurofilament light concentrations reflect neuronal damage in systemic lupus Erythematosus. BMC Neurol..

[46] Strehlow F., Bauer S., Martus P., Weller M., Roth P., Schlegel U., Seidel S., Scheibenbogen C., Korfel A., Kreher S. (2016). Osteopontin in cerebrospinal fluid as diagnostic biomarker for central nervous system lymphoma. J. Neurooncol..

[47] Hirohata S., Miyamoto T. (1990). Elevated levels of interleukin-6 in cerebrospinal fluid from patients with systemic lupus erythematosus and central nervous system involvement. Arthritis Rheum..

[48] Chazal T., Costopoulos M., Maillart E., Fleury C., Psimaras D., Legendre P., Pineton de Chambrun M., Haroche J., Lubetzki C., Amoura Z. (2019). The cerebrospinal fluid CD4/CD8 ratio and interleukin-6 and -10 levels in neurosarcoidosis: A multicenter, pragmatic, comparative study. Eur. J. Neurol..

[49] Levraut M., Landes C., Mondot L., Cohen M., Bresch S., Brglez V., Seitz-Polski B., Lebrun-Frenay C. (2022). Kappa Free Light Chains, Soluble Interleukin-2 Receptor, and Interleukin-6 Help Explore Patients Presenting With Brain White Matter Hyperintensities. Front. Immunol..

[50] Ichinose K., Arima K., Ushigusa T., Nishino A., Nakashima Y., Suzuki T., Horai Y., Nakajima H., Kawashiri S.Y., Iwamoto N. (2015). Distinguishing the cerebrospinal fluid cytokine profile in neuropsychiatric systemic lupus erythematosus from other autoimmune neurological diseases. Clin. Immunol..

[51] Martynova E., Goyal M., Johri S., Kumar V., Khaibullin T., Rizvanov A.A., Verma S., Khaiboullina S.F., Baranwal M. (2020). Serum and Cerebrospinal Fluid Cytokine Biomarkers for Diagnosis of Multiple Sclerosis. Mediat. Inflamm..

[52] Alvarez E., Piccio L., Mikesell R.J., Klawiter E.C., Parks B.J., Naismith R.T. (2013). Cross AH. CXCL13 is a biomarker of inflammation in multiple sclerosis, neuromyelitis optica, and other neurological conditions. Mult. Scler..

[53] Leth T.A., Dessau R.B., Møller J.K. (2022). Discriminating between Lyme neuroborreliosis and other central nervous system infections by use of biomarkers CXCL13 and IL-6. Ticks Tick Borne Dis..

[54] Hinsinger G., Galéotti N., Nabholz N., Urbach S., Rigau V., Demattei C., Lehmann S., Camu W., Labauge P., Castelnovo G. (2015). Chitinase 3-like proteins as diagnostic and prognostic biomarkers of multiple sclerosis. Mult. Scler..

[55] Kušnierová P., Zeman D., Hradílek P., Zapletalová O., Stejskal D. (2020). Determination of chitinase 3-like 1 in cerebrospinal fluid in multiple sclerosis and other neurological diseases. PLoS ONE.

[56] Lucchini M., De Arcangelis V., Piro G., Nociti V., Bianco A., De Fino C., Di Sante G., Ria F., Calabresi P., Mirabella M. (2023). CSF CXCL13 and Chitinase 3-like-1 Levels Predict Disease Course in Relapsing Multiple Sclerosis. Mol. Neurobiol..

[57] Aslam N., Khan I.U., Bashamkh A., Alghool F.A., Aboulnour M., Alsuwayan N.M., Alturaif R.K., Brahimi S., Aljaamel S.S., Al Khamdi K. (2022). Multiple Sclerosis Diagnosis Using Machine Learning and Deep Learning: Challenges and Opportunities. Sensors.

