# Supplementary material for: Cerebrospinal Fluid Biomarkers in Differential Diagnosis of Multiple Sclerosis and Systemic Inflammatory Diseases with Central Nervous System Involvement
Source: Biomedicines. 2023 Feb 1;11(2):425. doi: 10.3390/biomedicines11020425 (PMC9953577; doi:10.3390/biomedicines11020425)
Supplement: Supplementary file 1 [file biomedicines-11-00425-s001.zip › biomedicines-2147805-supplementary.pdf]

Supplementary Materials

Cerebrospinal Fluid Biomarkers in Differential Diagnosis of Multiple Sclerosis and Systemic Inflammatory Diseases with Central Nervous System Involvement

Mariola Świderek-Matysiak , Magdalena Oset, Małgorzata Domowicz, Grażyna Galazka, Magdalena Namiecińska and Mariusz Stasiołek

Table S1. The catalog/lot number of used ELISA kits.

NF-light	UmanDiagnostics, Sweden, Umea	cat: 10-7001	LOT: 70736, 70757, 70784, 70804
GFAP	EIAab, China	cat: E0068h	LOT: 0D135C, 0L155C, 0F195C, 0I225C
IL6	Biorbyt, UK	cat: orb50052	LOT: B2984
IFN gamma	Biorbyt, UK	cat: orb50080	LOT: B2969
CXCL13	Biorbyt, UK	cat: orb890880	LOT: CB6714
OPN	Biorbyt, UK	cat: orb50093	LOT: B3060
YKL-40	Biorbyt, UK	cat: orb219580	LOT: ER2487

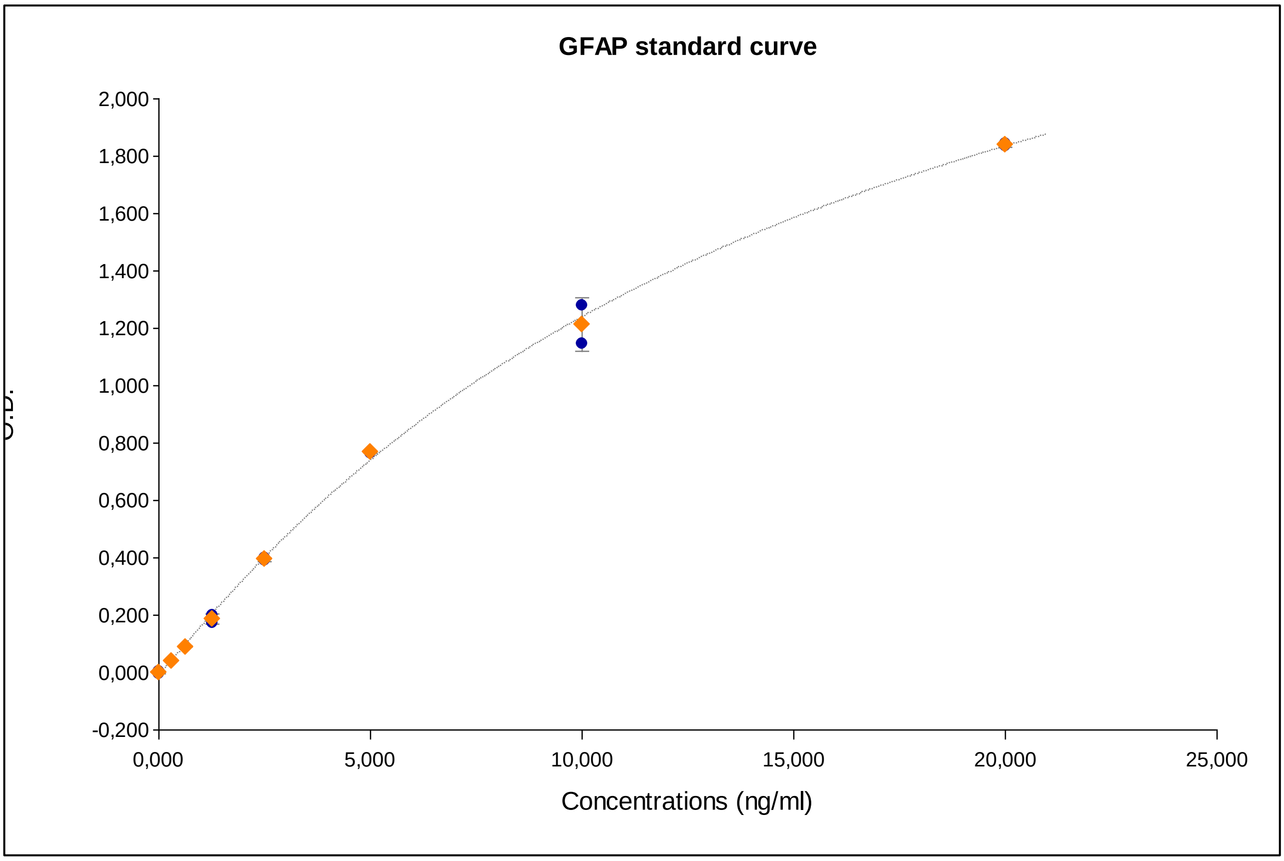

GFAP standard curve
O.D.
2,000
1,800
1,600
1,400
1,200
1,000
0,800
0,600
0,400
0,200
0,000
-0,200
0,000
5,000
10,000
15,000
20,000
25,000
Concentrations (ng/ml)

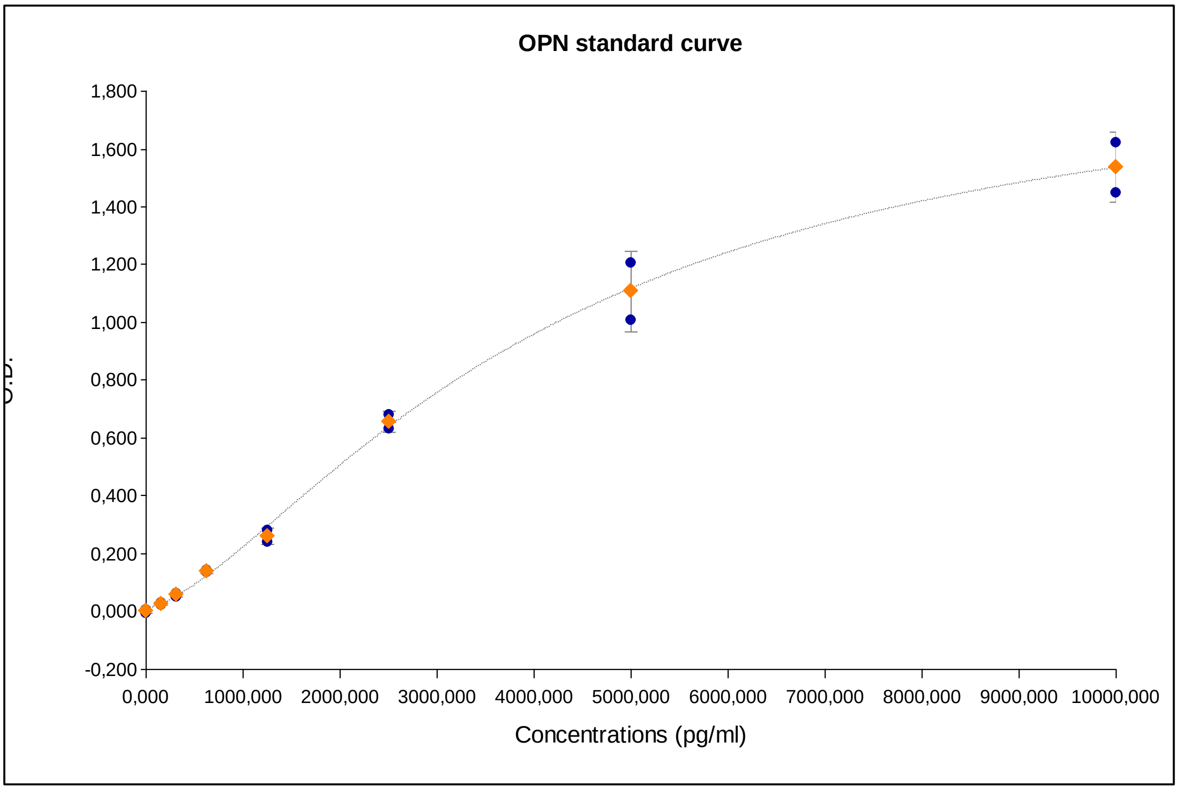

OPN standard curve
O.D.
1,800
1,600
1,400
1,200
1,000
0,800
0,600
0,400
0,200
0,000
-0,200
0,000
1000,000
2000,000
3000,000
4000,000
5000,000
6000,000
7000,000
8000,000
9000,000
10000,000
Concentrations (pg/ml)

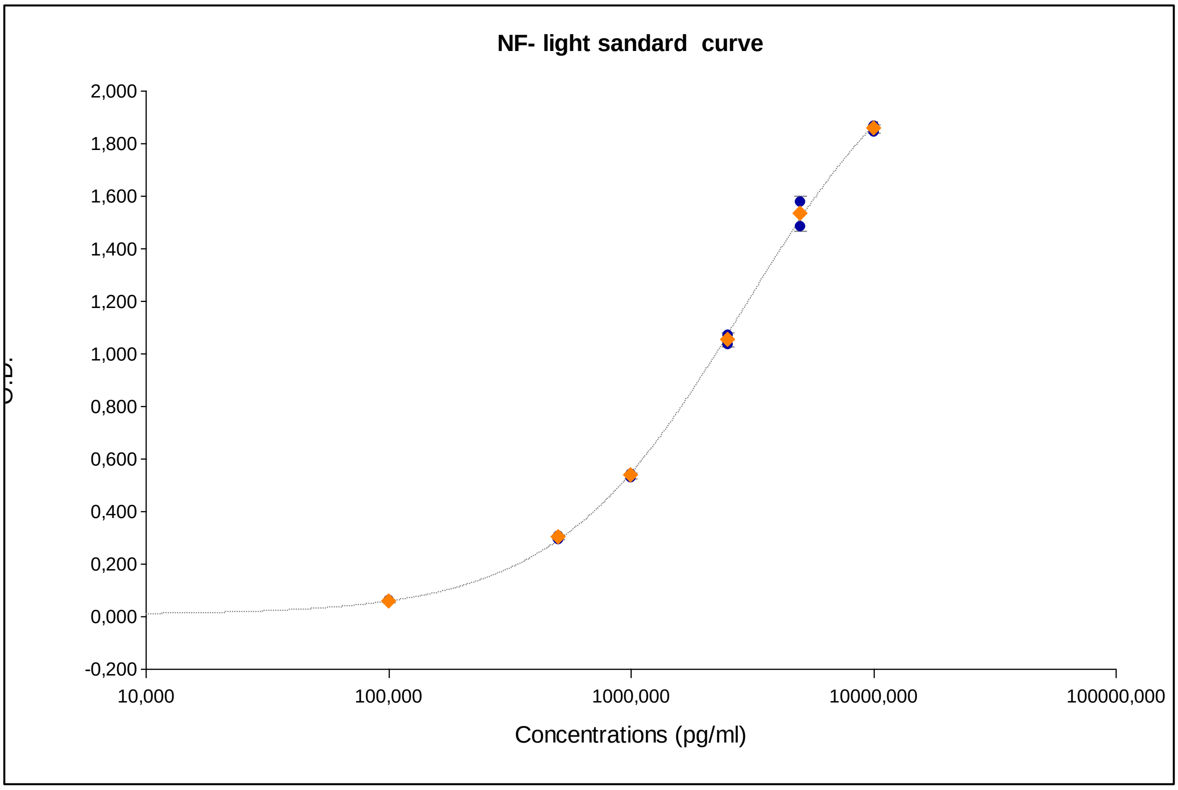
NF- light sandard curve
O.D.
2,000
1,800
1,600
1,400
1,200
1,000
0,800
0,600
0,400
0,200
0,000
-0,200
10,000
100,000
1000,000
10000,000
100000,000
Concentrations (pg/ml)

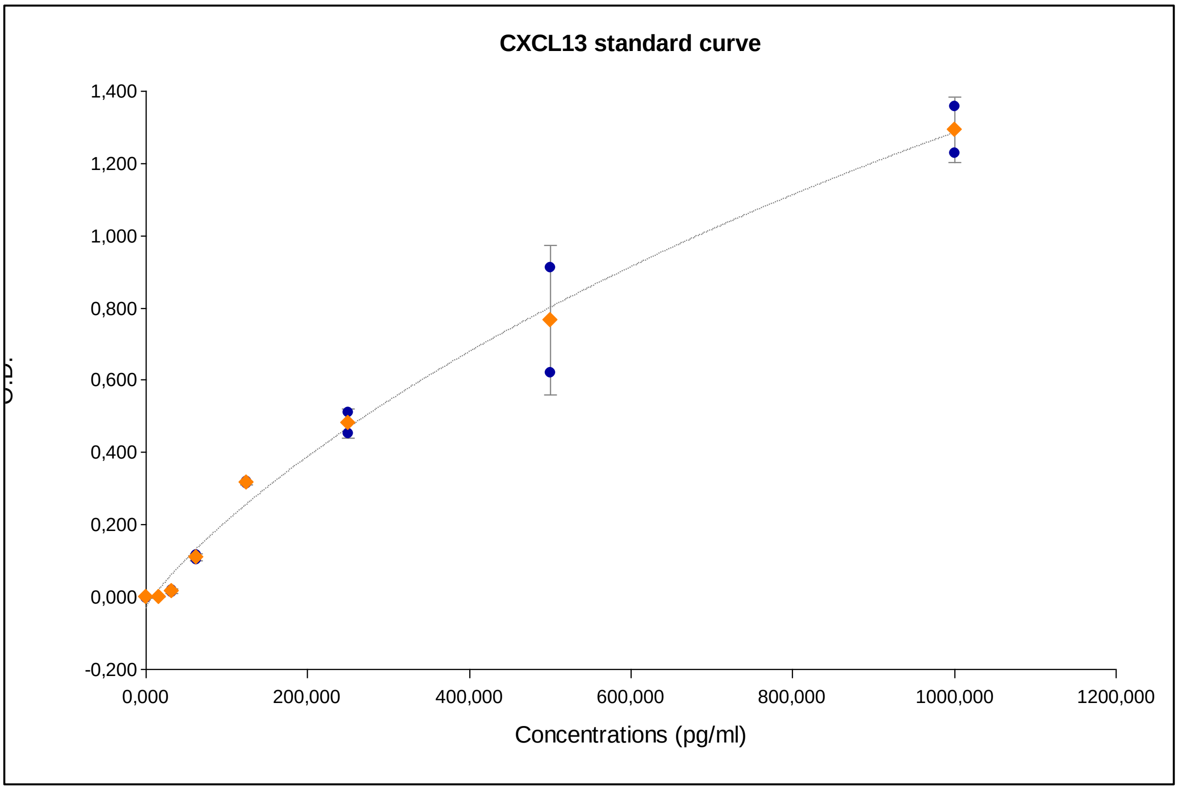
CXCL13 standard curve
O.D.
1,400
1,200
1,000
0,800
0,600
0,400
0,200
0,000
-0,200
0,000
200,000
400,000
600,000
800,000
1000,000
1200,000
Concentrations (pg/ml)

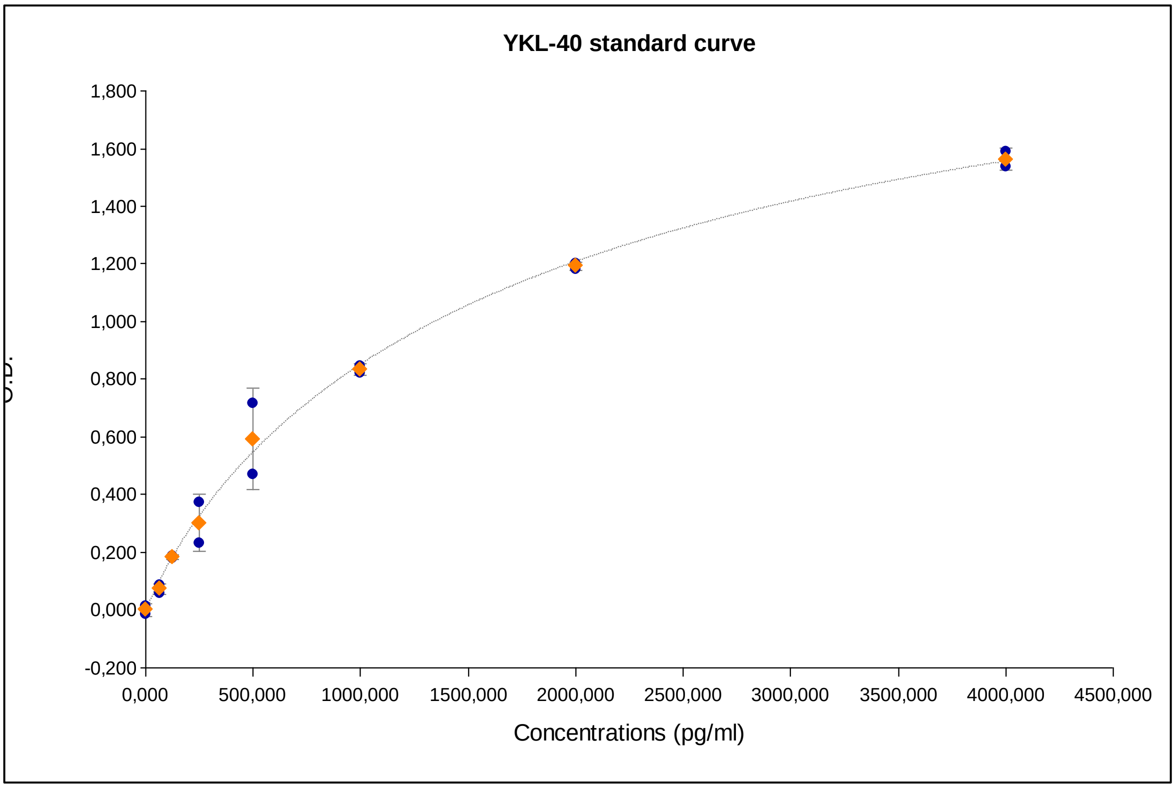
YKL-40 standard curve
O.D.
1,800
1,600
1,400
1,200
1,000
0,800
0,600
0,400
0,200
0,000
-0,200
0,000
500,000
1000,000
1500,000
2000,000
2500,000
3000,000
3500,000
4000,000
4500,000
Concentrations (pg/ml)

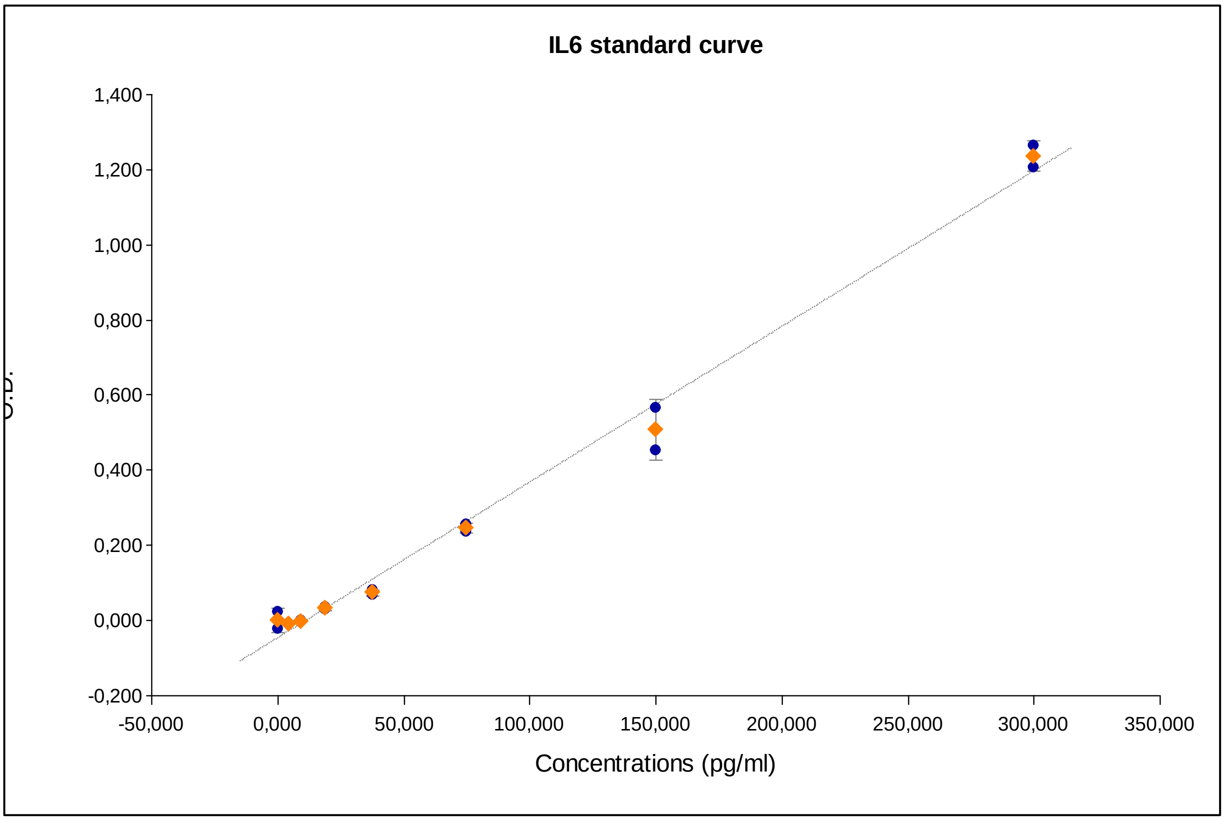
IL6 standard curve
O.D.
1,400
1,200
1,000
0,800
0,600
0,400
0,200
0,000
-0,200
-50,000
0,000
50,000
100,000
150,000
200,000
250,000
300,000
350,000
Concentrations (pg/ml)

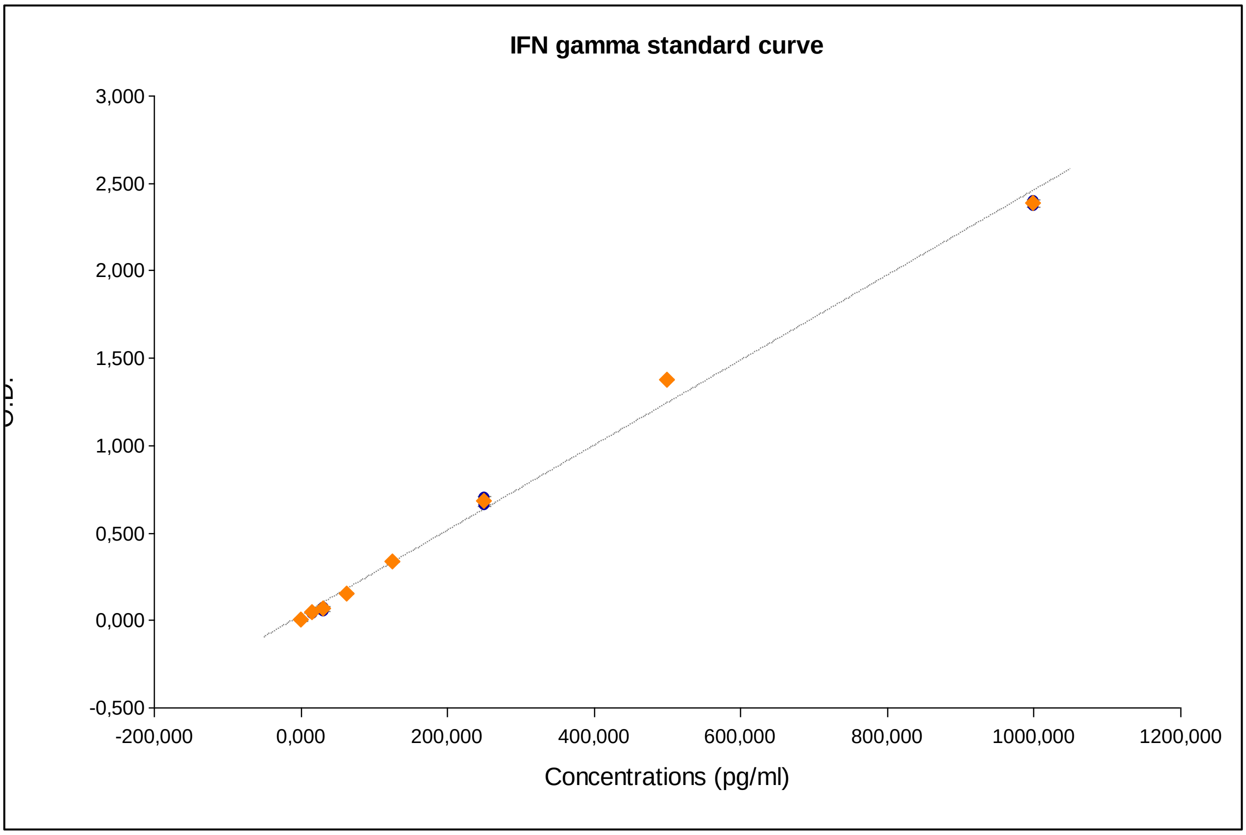

Figure S1. The representative standard curves of used ELISA kits.
